# Pulling Forces Differentially Affect Refolding Pathways Due to Entangled Misfolded States in SARS-CoV-1 and SARS-CoV-2 Receptor Binding Domain

**DOI:** 10.3390/biom14101327

**Published:** 2024-10-18

**Authors:** Pham Dang Lan, Edward P. O’Brien, Mai Suan Li

**Affiliations:** 1 Institute for Computational Sciences and Technology, Ho Chi Minh City 71506, Vietnam; l72mss@gmail.com; 2 Faculty of Physics and Engineering Physics, VNUHCM-University of Science, 227, Nguyen Van Cu Street, District 5, Ho Chi Minh City 72700, Vietnam; 3 Department of Chemistry, Pennsylvania State University, University Park, PA 16802, USA; epo2@psu.edu; 4 Bioinformatics and Genomics Graduate Program, The Huck Institutes of the Life Sciences, Pennsylvania State University, University Park, PA 16802, USA; 5 Institute for Computational and Data Sciences, Pennsylvania State University, University Park, PA 16802, USA; 6 Institute of Physics, Polish Academy of Sciences, 02-668 Warsaw, Poland

**Keywords:** protein folding, SARS-CoV-2 RBD, SARS-CoV-1 RBD, quenched force, lasso entanglement, folding pathways

## Abstract

Single-molecule force spectroscopy (SMFS) experiments can monitor protein refolding by applying a small force of a few piconewtons (pN) and slowing down the folding process. Bell theory predicts that in the narrow force regime where refolding can occur, the folding time should increase exponentially with increased external force. In this work, using coarse-grained molecular dynamics simulations, we compared the refolding pathways of SARS-CoV-1 RBD and SARS-CoV-2 RBD (RBD refers to the receptor binding domain) starting from unfolded conformations with and without a force applied to the protein termini. For SARS-CoV-2 RBD, the number of trajectories that fold is significantly reduced with the application of a 5 pN force, indicating that, qualitatively consistent with Bell theory, refolding is slowed down when a pulling force is applied to the termini. In contrast, the refolding times of SARS-CoV-1 RBD do not change meaningfully when a force of 5 pN is applied. How this lack of a Bell response could arise at the molecular level is unknown. Analysis of the entanglement changes of the folded conformations revealed that in the case of SARS-CoV-1 RBD, an external force minimizes misfolding into kinetically trapped states, thereby promoting efficient folding and offsetting any potential slowdown due to the external force. These misfolded states contain non-native entanglements that do not exist in the native state of either SARS-CoV-1-RBD or SARS-CoV-2-RBD. These results indicate that non-Bell behavior can arise from this class of misfolding and, hence, may be a means of experimentally detecting these elusive, theoretically predicted states.

## 1. Introduction

The effect of non-covalent lasso entanglements on protein folding kinetics in solution [1,2] and on ribosomes [3,4] has recently been an area of active research. Non-covalent lasso entanglements are formed when a segment of the protein backbone forms a loop closed by a native contact between two residues and is threaded one or more times by the remaining N- or C-terminal backbone segment. The formation of these entanglements as both on- and off-pathway intermediates was found to affect folding times and folding pathways. Experimentally, a means of modulating the kinetics of protein folding is by applying force to the terminal ends of proteins [5,6]. It would, therefore, be interesting to study whether external force can interplay with the effect of lasso entanglements as a potential means to detect the presence of this class of heterogeneous misfolding. For illustration, here we study the folding of the receptor binding domain (RBD) of SARS-CoV-1 and SARS-CoV-2. SARS-CoV is the abbreviation for severe acute respiratory syndrome coronavirus, and SARS-CoV-1 caused a global epidemic in several countries in 2002 and 2003, while SARS-CoV-2 is the strain of coronavirus that caused the COVID-19 pandemic.

Despite approximately 80% sequence similarity [7], SARS-CoV-2 has been shown to bind to the hACE2 (human angiotensin-converting enzyme 2) receptor more effectively than SARS-CoV-1 [8,9], which plays a crucial role in the explanation of its high transmission and infection rates during COVID-19 pandemic. Atomic-level structural analysis revealed that altered residues in the SARS-CoV-2 RBD result in better fitness and improved interactions of RBD with ACE2 [10]. There are also differences in the electrostatic potential characteristics on the RBD surfaces. Lys417, which is absent in the SARS-CoV-1 RBD, promotes an expanded positive charge on the SARS-CoV-2 RBD compared to the SARS-CoV-1 RBD, leading to increased binding affinity of the SARS-CoV-2 RBD to ACE2 [11]. This was confirmed by molecular dynamics simulations, which demonstrated that electrostatic interactions drive the interaction between RBDs and ACE2 [12].

**Figure 1:**
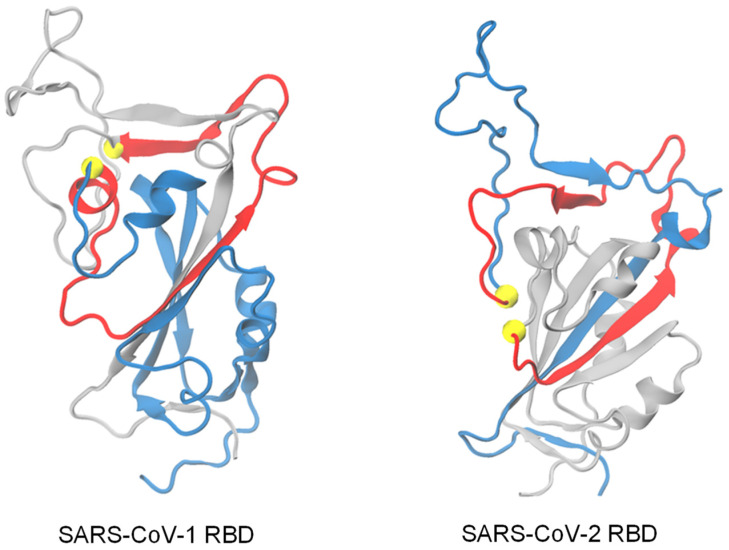
Representative native entanglements in the native structures of SARS-CoV-1 RBD and SARS-CoV-2 RBD. These structures were obtained using AlphaFold2 and MD simulations. The loops (colored red) are closed by native contacts (colored yellow). (**Left**) The loop is closed by the native contact between residue 99 and 136, with both N and C termini threading through the loop. Note that only the C-terminal thread is colored blue for more visibility. (**Right**) The loop is closed by the native contact between residue 108 and 146, with the C-terminal thread highlighted in blue.

On the other hand, modifications in protein sequence can be expected to result in differences in the folding kinetics of SARS-CoV-1 and SARS-CoV-2 RBDs, so the way they fold may also affect their binding abilities. In this study, we used coarse-grained molecular dynamics simulations to model and compare the refolding processes of SARS-CoV-1 RBD and SARS-CoV-2 RBD starting from unfolded conformations. The study of RBD folding becomes more interesting by the presence of non-covalent lasso entanglements in their native state topology (Figure 1). Observations of the folding process of some knot proteins have shown that the threading of the loop is a rate-limiting step with a high barrier [13,14] and can slow down the folding of knot proteins. The existence of non-covalent lasso entanglements in the native structure, which may lead to the appearance of off-pathway misfolded states [3,4,15], is expected to cause a similar effect on the folding of this class of proteins. These misfolded states, although similar in structure to the native state, contain non-native entanglements and are predicted to be able to, in some cases, bypass the proteostasis machinery [16], remaining soluble but potentially reducing the amount of functional protein or its ability to interact with partners [3,17].

To mimic SMFS refolding experiments and the force exerted on the protein through interactions with surrounding cellular molecules during the folding process, a constant force of 5 pN was applied to the RBD termini in our simulations. In SMFS experiments, a force of a few pN (~2 pN–8 pN) was used, and at this force, proteins can refold. Therefore, our choice of a force of 5 pN is a standard value, and folding events can be observed in our simulations. We find that the folding of SARS-CoV-2 RBD is sensitive to the external force, as indicated by the presence of unfolded sampled trajectories at the end of MD simulations. The estimated folding time significantly increased compared to the zero-force case. The SARS-CoV-1 RBD was insensitive to the applied force, as evidenced by a similar number of sampled trajectories capable of folding and comparable folding times with or without force. Our simulation results indicate that although off-pathway intermediates are common in the folding of both RBDs, the presence of force helps prevent the formation of misfolded entanglements in SARS-CoV-1 RBD, thereby avoiding kinetic trapping into misfolded states, promoting folding and offsetting the expected force-induced slowdown in proteins that fold in a two-state manner.

## 2. Materials and Methods

### 2.1. Sequences of SARS-CoV-1 and SARS-CoV-2 RBDs

The amino acid sequence of RBDs was obtained from the full-length sequences of SARS-CoV-1 (ID: cd21481) and SARS-CoV-2 (ID: cd21480) spike proteins [18]. The SARS-CoV-1 RBD includes residues 306–527, while SARS-CoV-2 RBD spans residues 319–541. The aligned sequences of these two RBDs using a sequence analysis tool [19] show 82.1% similarity and 73.1% identity.

### 2.2. Prediction of SARS-CoV-1 RBD and SARS-CoV-2 RBD Structures

The SARS-CoV-1 RBD and SARS-CoV-2 RBD structures were predicted using AlphaFold2 [20] via the COSMIC^2^ platform [21]. Three 500 ns MD simulations were conducted to equilibrate the predicted structures to identify stable RBD structures using the cluster method (see below).

### 2.3. Molecular Dynamics (MD) Simulation

The proteins were modeled using the FF14SB force field [22] and solvated in a cubic box of TIP3P water molecules [23] with the box boundary at least 12 Å away from any protein atoms. The system was neutralized with Na^+^ and Cl^−^ counterions, and 0.15 M sodium chloride was added to simulate the cellular salt concentration. These steps were prepared using CHARMM-GUI [24].

The simulations were performed using the GROMACS 2020.5 package [25]. Periodic boundary conditions were applied, and long-range electrostatic interactions were calculated using the particle mesh Ewald method with a nonbonded cutoff distance of 9 Å. Bond lengths were constrained using the LINCS algorithm [26], and an integration step of 2 fs was used. The energy of the system was minimized using the steepest-descent algorithm, then equilibrated in the NVT and NPT ensembles at 300 K for 1 ns each with protein position restraints of 10 kcal/mol Å^−2^. An additional 1 ns NPT run with protein position restraints reduced to 1 kcal/mol Å^−2^ was performed before production runs. A Nose–Hoover thermostat [27] and Parrinello–Rahman barostat [28] were used to maintain the temperature and pressure at 300 K and 1 atm.

### 2.4. Coarse-Grained Model and Details of Refolding Simulation

Simulating the folding process by all-atom MD, especially in the presence of an external force, using all-atom models is challenging due to limited computational resources. Therefore, we use a previously published Gō-based coarse grain methodology that favors the native state by distinguishing between native and non-native interactions [3,4,29]. In this model, each amino acid is represented by a single interaction site located at the C*_α_* coordinates and assigned a specific van der Waals radius. The potential energy of a given configuration is computed using the following equation:

(1) $$\begin{array}{l} E=\sum_{i}k_{b}{\left(r_{i}-r_{0}\right)}^{2}+\sum_{i}\sum_{j}^{4}k_{\varphi ,ij}\left[1+\cos\left(j\varphi_{i}-\delta_{ij}\right)\right]\\ \;+\sum_{i}-\frac{1}{\gamma }ln\left\{exp\left[-\gamma \left(k_{\alpha }{\left(\theta_{i}-\theta_{\alpha }\right)}^{2}+\varepsilon_{\alpha }\right)\right]+exp\left[-\gamma k_{\beta }{\left(\theta_{i}-\theta_{\beta }\right)}^{2}\right]\right\}+\sum_{ij}\frac{q_{i}q_{j}e^{2}}{4\pi \varepsilon_{0}\varepsilon_{r}r_{ij}}\exp\left[-\frac{r_{ij}}{l_{D}}\right]\\ \;+\sum_{ij\;\in \;\left\{NC\right\}}\varepsilon_{ij}^{NC}\left[13{\left(\frac{\sigma_{ij}}{r_{ij}}\right)}^{12}-18{\left(\frac{\sigma_{ij}}{r_{ij}}\right)}^{10}+4{\left(\frac{\sigma_{ij}}{r_{ij}}\right)}^{6}\right]\\ \;+\sum_{ij\notin \left\{NC\right\}}\varepsilon_{ij}^{NN}\left[13{\left(\frac{\sigma_{ij}}{r_{ij}}\right)}^{12}-18{\left(\frac{\sigma_{ij}}{r_{ij}}\right)}^{10}+4{\left(\frac{\sigma_{ij}}{r_{ij}}\right)}^{6}\right]. \end{array}$$

This is the sum of contributions from C_α_−C_α_ virtual bonds, dihedral angles, bond angles, electrostatic interactions, Lennard–Jones-like native interactions, and repulsive non-native interactions, respectively. The parameters of the model are described elsewhere [30].

Based on the native structure of RBD determined from all-atom MD simulations, the total number of native contacts, $N_{native}$, was calculated by counting all pairs of residues within secondary structure elements that are at least 4 residues apart and have a distance of 8 Å or less between their C_α_. Any conformation k is compared to the native conformation through the fraction of native contacts, *Q*, defined as

(2) $$Q=\frac{N_{k}}{N_{native}},$$

where $N_{k}$ represents the number of residue pairs that form contacts matching the elements of the $N_{native}$ list. The stability of the coarse-grained model of RBD proteins was evaluated by selecting an appropriate scaling factor η for Lennard–Jones interactions of all native contacts based on the previous training set [31]. A protein model is considered stable if the fraction of native contacts, *Q*, calculated from all simulation snapshots exceeds 0.69 for at least 98%. For this purpose, each tested η value is multiplied by the $\varepsilon_{ij}^{NC}$ term in Equation (1) and three 1 μs MD simulations, each initiated from the native structure, were performed. An η of 1.4213 was selected based on this procedure for RBDs.

One hundred unfolding MD simulations, each 100 ns long, were performed at 1000 K, starting from a representative structure to generate unfolded structures for subsequent refolding MD simulations. To ensure a fully unfolded conformation, 100 conformations with the largest end-to-end distance and no native contact remaining at the end of 100 unfolded simulations were selected. In our MD simulation protocol, the initial structures were first aligned along the *x*-axis, and an external force applied to the two terminal atoms was implemented in the x-direction. All simulations were conducted using Langevin dynamics at 310 K with OpenMM 7.7 [32], and an integration time step of 0.015 ps and a friction coefficient of 0.05 ps^−1^ were used.

### 2.5. Determining Folded Trajectories and Calculating the Folding Time

A given protein trajectory is considered to be folded when the mode of *Q* values, *Q*_mode_, calculated over a sliding 15 ns window of a refolding simulation time series, is greater than or equal to the threshold value *Q*_threshold_. To determine *Q*_threshold_, ten 1 μs coarse-grained MD simulations were performed at 310 K, each initialized from the native-state structure. *Q*_threshold_ is calculated as *Q*_threshold_ = $\langle Q_{mode}^{NS}\rangle$−3*σ*, where $\langle Q_{mode}^{NS}\rangle$ is the average *Q*_mode_ over a sliding 15 ns of ten 1 μs native state simulations, and *σ* is the standard deviation [3]. The threshold *Q* values for RBD proteins are listed in Table 1.

Based on this definition, the folding time was estimated as the median first passage time of folded trajectories in a total of 100 simulation trajectories that were run. If the fraction of folded trajectories is less than 50%, we calculate the survival probability of the unfolded state $S_{U}\left(t\right)$ as a function of time, then fit to a double exponential equation $S_{U}\left(t\right)=f_{1}\exp\left(-k_{1}t\right)+f_{2}\exp\left(-k_{2}t\right)$, where $f_{1}+f_{2}=1$. This fitting equation corresponds to a kinetic scheme that assumes the unfolded states follow two distinct parallel folding pathways toward the folded state [4]. The folding times of the two kinetic phases are calculated as follows: $\tau_{1}=1/k_{1}$ and $\tau_{2}=1/k_{2}$, and the larger of them determines the overall time scale of the folding process.

### 2.6. Identification of Entanglements and Characterization of Misfolded Structure Through Changes in Entanglement

Here, we used Gaussian linking numbers defined through double Gauss integrals [33] with modifications to detect lasso-like entanglements between a closed contact loop and open terminal tails in coarse-grain protein structure [1,34]. The loop is closed by connecting residues i and j that form a native contact, and the entanglement of each N- or C-terminal tail with the loop is characterized by partial linking numbers denoted $g_{N}$ and $g_{C}$. For a given structure of an N-residue protein with a native contact present at residues (i, j), the linking numbers $g_{N}\left(i,\;j\right)$ and $g_{C}\left(i,\;j\right)$ were calculated as

(3) $$\left\{\begin{array}{c} g_{N}\left(i,\;j\right)=\frac{1}{4\pi }\sum_{m=6}^{i-5}\sum_{n=i}^{j-1}\frac{R_{m}-R_{n}}{{\left|R_{m}-R_{n}\right|}^{3}}\cdot \left(dR_{m}\times dR_{n}\right)\;\\ g_{C}\left(i,\;j\right)=\frac{1}{4\pi }\sum_{m=i}^{j-1}\sum_{n=j+4}^{N-6}\frac{R_{m}-R_{n}}{{\left|R_{m}-R_{n}\right|}^{3}}\cdot \left(dR_{m}\times dR_{n}\right) \end{array}\right.,$$

where the first 5 residues of the N-terminal curve, the last 5 residues of the C-terminal curve and the 4 residues before and after the native contact are excluded to eliminate the error introduced by both the high flexibility and contiguity of the termini and trivial entanglements in the local structure [35]. The coordinates $R_{l}$ and the gradient $dR_{l}$ of the point l on the curves in Equation (3) were calculated as

(4) $$\left\{\begin{array}{c} R_{l}=\frac{1}{2}\left(r_{l}+r_{l+1}\right)\\ dR_{l}=r_{l+1}-r_{l}\; \end{array}\right.,$$

where $r_{l}$ is the coordinates of the C_α_ atom in residue l.

The total linking number for a native contact (i, j) is therefore estimated as

(5) $$g\left(i,j\right)=\mathrm{round}\left(\left|g_{N}\left(i,\;j\right)\right|\right)+\mathrm{round}\left(\left|g_{C}\left(i,j\right)\right|\right).$$

Comparing the total linking number for a native contact (i, j) with the number of links in the native state allows us to detect gain or loss of linking between the backbone trace loop and the terminal open curves, as well as any switches in chirality [3]. For a given conformation with $N_{k}$ native contacts formed, where $N_{k,∆}$ of them ($N_{k,∆}\leq N_{k}$) changed in entanglement (gain or loss) compared to the native, the fraction of native contacts with a change in entanglement, *G*, is calculated as

(6) $$G=\frac{N_{k,∆}}{N_{native}}.$$

A combination of (*Q*, *G*) parameters was used to characterize misfolded conformations.

### 2.7. Identification of Folding Pathways

Entangled states can form kinetic traps with large energy barriers that may be hidden when the conformation space is projected onto *Q* alone. Therefore, we derive a log probability surface as a function of (*Q*, *G*) to characterize the conformation space.

To infer folding pathways from simulated trajectories, we first used the K-mean++ algorithm with 300 clusters to identify microstates from the (*Q*, *G*) pool of simulation data, then refined the resulting clusters into a small number of metastable states using PCCA+ algorithms. Finally, the metastable states were used to interpret the folding pathways using the protocol described in [15].

## 3. Results and Discussions

### 3.1. Determination of Structure of SARS-CoV-1 and SARS-CoV-2 RBDs

**Figure 2:**
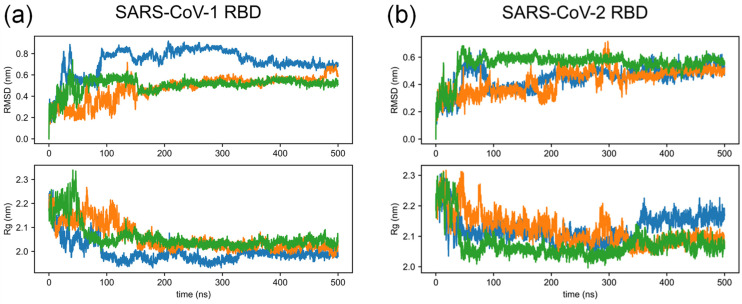
The root mean square deviation (RMSD) and the radius of gyration (Rg) of the backbone from three 500 ns all-atom MD simulations for (**a**) SARS-CoV-1 RBD and (**b**) SARS-CoV-2 RBD.

Three individual trajectories, each being a 500 ns all-atom MD run, were performed for SARS-CoV-1 and SARS-CoV-2 RBDs starting from the structures predicted by AlphaFold2 (pLDDT scores are 89.4 and 88.1, respectively). Details of the MD simulation are described in Section 2.3. Figure 2 shows the time dependence of RMSD and R_g_ of RBDs, which shows that the system is stabilized at approximately 400 ns. Snapshots from the last 100 ns (400 ns–500 ns) of all three trajectories were collected and clustered based on the RMSD matrix using the GROMOS method [36] implemented in GROMACS with an RMSD cutoff of 0.2 nm. The representative conformation from the most populated cluster was selected as the reference structure or native structure of SARS-CoV-1 and SARS-CoV-2 RBDs for the refolding study.

### 3.2. SARS-CoV-2 RBD Folds More Effectively Than SARS-CoV-1 RBD

**Figure 3:**
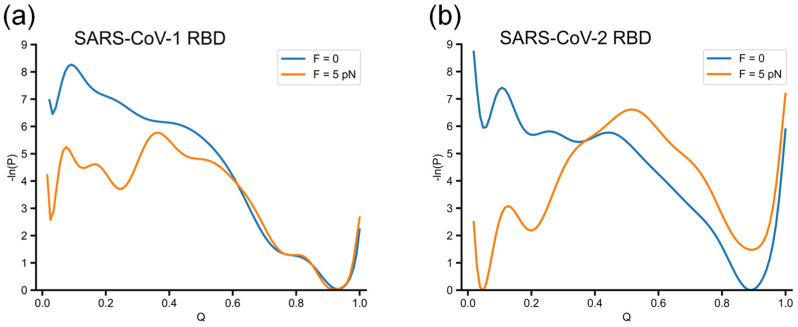
Dependence of −ln(*P*) on *Q*, where *P* is the probability of sampling a particular *Q* value of (**a**) SARS-CoV-1 RBD and (**b**) SARS-CoV-2 RBD. An additional minimum is observed at *Q*~0.2, which occurs in the presence of the external force.

To explore the folding process of RBDs, we computed log probability landscapes from the distribution of parameter *Q* collected from 100 simulated trajectories. Due to high sequence identity and similar native structures, refolding from the thermally unfolded ensemble of SARS-CoV-1 RBD and SARS-CoV-2 RBD is largely similar, including highly disordered initial conformations at *Q*~0.05 and more stable conformations at *Q*~0.9 (blue curves, Figure 3a,b). SARS-CoV-1 RBD rapidly collapsed into globular conformations (*Q* between 0.8 and 1.0) near the stable native state, while SARS-CoV-2 RBD sampled more conformations at *Q*~0.2 and 0.4 before reaching its native state.

To further characterize the difference in folding of SARS-CoV-1 RBD and SARS-CoV-2 RBD, we identified folded trajectories from refolding simulations and calculated folding times as described in the Methods section. The results are shown in Table 1. The percentage of trajectories out of a total of 100 simulated trajectories that successfully folded to the native states after 6 μs simulations is 56% for SARS-CoV-1 RBD and 97% for SARS-CoV-2 RBD, indicating that the refolding from the unfolded state of SARS-CoV-2 RBD is more effective. SARS-CoV-2 RBD also folded slightly faster, with an estimated folding time of 257.4 ns, compared to 369.0 ns for SARS-CoV-1 RBD. Our calculations of hydrophobicity based on the hydrophobicity scale of amino acids [37] showed that *H*_SARS-CoV-2 RBD_ = 64.99 kcal/mol is larger than *H*_SARS-CoV-1 RBD_ = 53.45 kcal/mol. This suggests the observed faster folding of SARS-CoV-2 RBD is biophysically reasonable because the higher the hydrophobicity, the faster the folding.

### 3.3. SARS-CoV-2 RBD Is More Sensitive to Force Than SARS-CoV-1 RBD

In vivo protein folding of proteins may differ from refolding in experiments in which an external force is exerted on unfolded proteins through protein–protein interactions [29,38,39,40,41,42]. A low force of a few pN can detect folding events during stretching–relaxing cycles in SMFS experiments [5,43,44,45,46,47,48,49,50,51]. Here, we explore the impact of quenched force on the refolding behavior of SARS-CoV-1 RBD and SARS-CoV-2 RBD by introducing a constant force of 5 pN applied to the N-terminus and C-terminus of RBDs in opposite directions during refolding simulations.

The log probability landscapes in the presence of force along the *Q* parameter are illustrated by the orange curves in Figure 3. Compared to the zero-force case, an additional minimum is observed at *Q*~0.2, which indicates a slowdown in folding due to the quenched force corresponding to the impact of force with both simulation datasets of SARS-CoV-1 RBD and SARS-CoV-2 RBD.

SARS-CoV-2 RBD is sensitive to the quenched force, as indicated by the high barrier in ln(*P*) at *Q*~0.5. In the presence of the pulling force, 58% of trajectories failed to fold by the end of the 6 μs simulations, compared to only 3% of trajectories in the absence of force. As expected, the folding time of SARS-CoV-2 RBD under a force of 5646 ns is much longer than the zero-force case, 257 ns. SARS-CoV-1 RBD is less sensitive to the external force, as shown by the lower barrier of ln(*P*) at *Q*~0.4. The difference in the percentage of folded trajectories between refolding without and with force is negligible: 56% and 58%, respectively. The estimated folding times of SARS-CoV-1 RBD are similar: 369.0 ns for *F* = 0 and 304.95 ns for *F* = 5 pN. The fact that the folding times are comparable for the zero and non-zero force cases, as shown in Section 3.4, is due to the ability of the external force to help the protein avoid some intermediate states.

### 3.4. Misfolded Intermediates with Altered Entanglement Are Present During Folding of SARS-CoV-1 RBD and SARS-CoV-2 RBD

**Figure 4:**
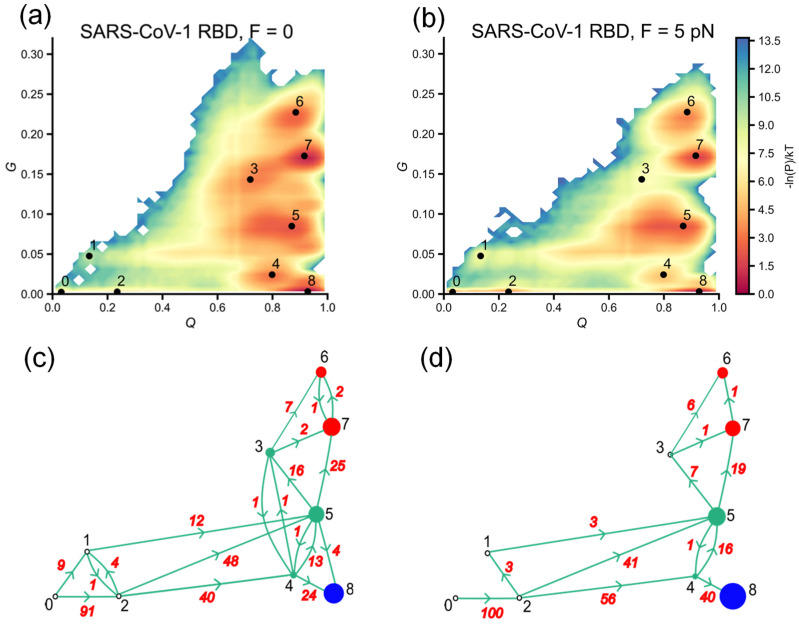
Misfolded states were observed during the folding of the SARS-CoV-1 RBD. Force may promote the folding process of the SARS-CoV-1 RBD, resulting in an increase in the percentage of trajectories that follow paths leading to correct folding. (**a**,**b**) represent −ln(*P*) surfaces, where *P* is the probability of sampling *Q* and *G* values during refolding simulations with *F* = 0 and *F* = 5 pN, respectively. (**c**,**d**) are transition networks of discrete trajectories along metastable states represented by nodes. Blue, green and red nodes correspond to folded, intermediate and misfolded states, respectively, that trajectories visited at the end of simulations. Red numbers indicate the number of transitions between states, and black numbers represent nodes.

As can be seen from the log probability landscape (Figure 3a), the SARS-CoV-1 RBD primarily sampled the conformational space along *Q* between 0.8 and 1.0, close to the native state in both simulations with *F* = 0 and *F* = 5 pN. However, approximately half of the 100 trajectories do not fold into the stable native structure. To further characterize the folding process, we constructed the log probability surface as a function of a combination of parameters *Q* and *G*, where *G* represents the change in entanglement as defined in the Methods section. As evident from the log probability surfaces (Figure 4a,b), a significant number of the sampled conformations are associated with intermediates states, namely 1 and 3–7, which can lead to misfolding due to the formation of native contacts with changes in entanglement. This likely accounts for the relatively large number of unfolded trajectories remaining at the end of the SARS-CoV-1 RBD simulations and suggests that misfolded states may be common in folding pathways.

Notably, states 5, 6 and 7, in which most native contacts are formed (*Q* > 0.8), have a significant change in entanglement, with more than 5% of native contacts containing non-native entanglements. Among intermediates states, state 7 populates a near-native state with *Q* values as high as the native state (*Q*~0.9) but with 15–20% of contacts containing non-native entanglements (*G*~0.15–0.2). This means that this state cannot be distinguished from the native state based on the parameter *Q* alone, although the fraction of contact *Q* is commonly used to determine whether a protein is folded or not. Moreover, the population of conformations sampled for state 7 is comparable to that of the native state.

**Figure 5:**
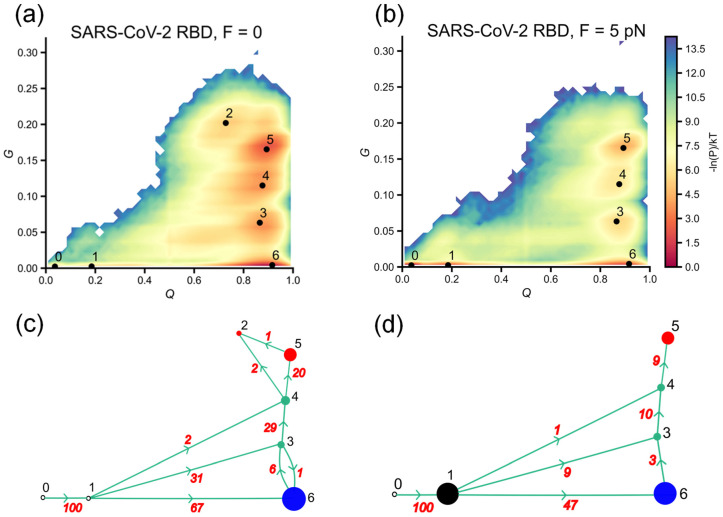
Misfolded states were also observed in the folding of the SARS-CoV-2 RBD. The force effect results in 43% of trajectories remaining in the unfolded state 1 at the end of the simulation. (**a**,**b**) represent the −ln(*P*) surfaces, where *P* is the probability of sampling certain values of *Q* and *G* from refolding simulations with *F* = 0 and *F* = 5 pN, respectively. (**c**,**d**) are transition networks of discrete trajectories along metastable states represented by nodes. Blue, green and red nodes correspond to folded, intermediate and misfolded states, respectively, that trajectories visited at the end of the simulations. The black node indicates that trajectories remain in the unfolded state 1 at the end of the simulations. Red numbers indicate the number of transitions between states, and black numbers represent nodes.

The existence of intermediate states with significant changes in entanglement is also common in the folding process of SARS-CoV-2 RBD. However, due to its sensitivity to force, SARS-CoV-2 RBD (Figure 5a,b) sampled a lower *G* conformation space compared to SARS-CoV-1 RBD (Figure 4a,b). As shown in Figure 5a,b, in addition to the native state (state 6), intermediate states 2–5 (*F* = 0) and 3–5 (*F* = 5 pN) are identified on (*Q*, *G*) log probability surfaces. Similar to state 7 on the log probability surfaces of SARS-CoV-1 RBD, state 5 exhibits characteristics of a near-native state, as it forms a large number of native contacts comparable to those of the native state (state 6) (*Q*~0.9–1.0). The ratio of contacts containing non-native entanglements (*G*~0.15–0.2) is similar to that of state 7 in SARS-CoV-1 RBD. However, the population of conformations corresponding to state 5 is smaller than that for state 6 in both simulations with and without external force. This indicates that the SARS-CoV-2 RBD still prefers sampling the native state and folds more efficiently compared to the SARS-CoV-1 RBD.

### 3.5. External Force Helps SARS-CoV-1 RBD to Explore More Efficiently Folding Pathways, Avoiding Misfolded States with Non-Native Entanglements

To characterize the role of intermediates and identify misfolded states in the folding process, the conformational space (*Q*, *G*) was clustered and assigned to metastable states to interpret the folding pathways, as described in the Methods section. The partitioning of the (*Q*, *G*) conformational space into metastable states is illustrated in Figures S1 and S2 for SARS-CoV-1 RBD and SARS-CoV-2 RBD, respectively.

As shown in Figure 4a and Figure 5a for the refolding pathways at zero force (*F* = 0), both SARS-CoV-1 RBD and SARS-CoV-2 RBD initially sampled the unfolded state region (*Q* < 0.2) before collapsing into globular conformations (*Q* > 0.7). In the unfolded region, SARS-CoV-1 RBD has the potential to form native contacts with non-native entanglements, as indicated by 13% of the trajectories following pathways that visited state 1 (*G*~0.05, Figure 4c). Overall, SARS-CoV-1 RBD follows two parallel pathways to intermediate states (2 → 4, 1|2 → 5 as numbered in Figure 4c): 40% of trajectories reach state 4, which is characterized by a lower *G* (*G* < 0.05), while 60% reach state 5, which has a higher *G* (*G*~0.05–0.1). Of the trajectories that sampled state 4, 24 out of 40 (60%) successfully folded to the native state. In contrast, trajectories that visited state 5 primarily advanced to states 6 and 7. States 6 and 7, which have the highest *G* (*G* > 0.15), are off-pathway misfolded states, as trajectories that visit these states tend to get trapped without any conversion out of these states (Figure 4c).

While SARS-CoV-1 RBD’s folding flux takes two distinct pathways to intermediates containing non-native entanglements, SARS-CoV-2 RBD refolding follows a single dominant pathway (67% of total trajectories) to correctly reach the folded state (state 6), while a less populated pathway leads to an intermediate state 3 with a *G* in the range of 0.05–0.1 (Figure 5c). Similar to what is observed for SARS-CoV-1 RBD (state 5), trajectories that visit intermediates with significant entanglement change (*G* > 0.05) and a high fraction of native contacts formed (*Q* > 0.8) then predominantly follow pathways leading to misfolded states (3 → 4 → 5|2). States 2 and 5 are off-pathway misfolded states, as the trajectories are kinetically trapped in these states.

A quenched force applied to the two ends of a protein can slow down the folding process by increasing the energy barrier to the formation of native contacts along the folding pathway. For an entangled protein such as the RBD, the presence of such a force might also be expected to disrupt the formation of native contacts that are essential to forming a non-native entanglement, which could lead to off-pathway intermediate states. For the SARS-CoV-1 RBD, despite the external force increasing the folding barriers (Figure 3a), 100% of the trajectories still sampled the high *Q* region in the conformation space after visiting the unfolded region characterized by states 1 and 2 (Figure 4a,c), implying that SARS-CoV-1 refolding is less sensitive to low force. However, the folding pathway diagrams (Figure 4c,d) suggest that the force improved the population of sampled trajectories that follow pathways leading to correct folding. In particular, 56% of the trajectories follow the 2 → 4 pathway, and 44% follow the 1|2 → 5 pathway, while in the zero-force case, these numbers are 40% and 60%, respectively. As the number of trajectories visiting state 4 (low *G*) increases, the percentage of sampled trajectories that successfully reach the folded state 5 in the force-induced refolding simulation (40%) is higher compared to the zero-force simulation (28%). Force also limited the formation of non-native entanglements at the early stage of refolding, as no trajectories were observed to follow the 0 → 1 pathway (Figure 4d).

**Figure 6:**
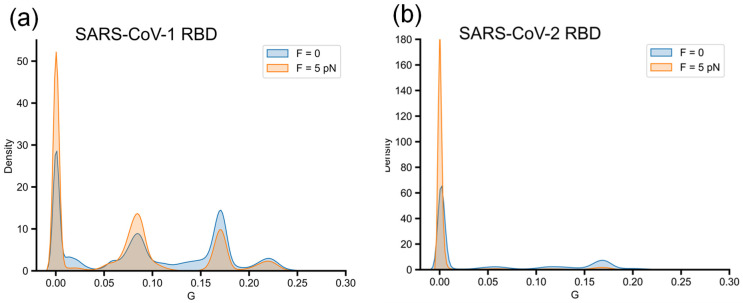
Conformational distribution along the *G* parameter of (**a**) SARS-CoV-1 RBD and (**b**) SARS-CoV-2 RBD with peaks are concise with characteristics of (*Q*, *G*) log probability surfaces, indicating the presence of structures with change in entanglement at different levels, namely *G*~0.05–0.1, 0.15–0.2, 0.2–0.25.

While the trajectories that sampled high *G* conformations tend to move toward misfolded states and appear to be kinetically trapped, there is a correlation between an increased probability of correctly folded trajectories and the higher number of lower-*G* conformations sampled in the folding behavior of SARS-CoV-1 RBD. As shown in Figure 6a, applying force causes a shift in the conformational population from higher *G* regions to lower *G* regions. Since 100 trajectories can reach globular conformations (high *Q*) at the end of the simulation, it is clear that the trajectories sampled more lower-*G* conformations in the presence of force. These results suggest that applying force helps prevent the formation of high-*G* conformations, thereby avoiding kinetic trapping into misfolded states and assisting the folding process of SARS-CoV-1 RBD correctly toward the native state.

For SARS-CoV-2 RBD, introducing a quenched force into simulations results in 43% of trajectories remaining in unfolded conformations (*Q*~0.2) at the end of simulations (state 1, Figure 5b,d), indicating that the folding process is sensitive to external force. Of the 57 trajectories that reach globular conformations (*Q* > 0.8), 47 trajectories (47% of the total simulation trajectories) then follow the correct folding pathway to reach folded state 6, as shown in the folding pathway diagram (Figure 5d). Notably, misfolded state 2, which has the highest *G* and is observed in the refolding simulation with *F* = 0 (Figure 5a), does not appear in the SARS-CoV-2 log probability landscape in the presence of force (Figure 5b). The population of conformations in the high *G* region (*G*~0.15–0.2) is reduced and offset by an increase in conformations sampled in the low *G* region (*G* < 0.05) due to the applied force (Figure 6b). It is unclear whether the force restricted the sampling of conformations with high *G,* leading to misfolded states in SARS-CoV-2 RBD since the force also increased the sampling of unfolded conformations (in the unfolded trajectories). 

## 4. Conclusions

Proteins refold slowly or inefficiently in vitro from denatured structures [52], suggesting that in vivo, translation elongation kinetics, trigger factor chaperones or post-translational quality controllers can affect the folding process of cellular proteins [29,38,53,54,55,56,57]. Here, using coarse-grained molecular dynamics simulations, we study the effect of a quenched force exerted through interactions with surrounding cellular molecules on the folding process of SARS-CoV-1 RBD and SARS-CoV-2 RBD, which contain non-covalent lasso entanglement motifs in their native structures.

The folding landscape of RBDs revealed that intermediates with changes in entanglement (compared to the native state) are popular in the folding pathways of both RBDs and tend to form misfolded states. The effect of external force is likely different for SARS-CoV-1 and SARS-CoV-2. Due to sensitivity to force, the folding time of SARS-CoV-2 increases significantly. In the presence of force, we hypothesize that force restricts the emergence of conformations to form contact with non-native entanglement while preventing the formation of native contacts at the same time. As a result, SARS-CoV-1 RBD sampled more conformations with less change in entanglement, and the population of sampled trajectories that follow pathways leading to correct folding was improved. Thus, the median folding time is comparable to the zero-force case. This supported one of the mechanisms by which chaperones facilitate folding by slowing down the folding process to avoid protein misfolding and aggregation [41,57,58].

Considering the change in entanglement of the protein structure during the folding process helps to discriminate the correct folded state from misfolded states, which are similar in structure to the native state but contain non-native entanglements. This is crucial for the determination of the folding kinetics of entangled proteins. The determination of the non-covalent lasso entanglement motif indicated that this entangled structure is common in the native state of proteins [59]. It will be interesting to explore whether the structural complexity of this class of protein, for example, deep or shallow entangled, correlates with the folding time.

## Figures and Tables

**Table 1:** Folding time and number of folded trajectories of SARS-CoV-1 RBD and SARS-CoV-2 RBD in simulations with and without force; *Q*_threshold_ values to determine folded trajectories were also included.

		*F* = 0		*F* = 5 pN	
Protein	*Q*_threshold_	Folded Trajectories	Folding Time (ns)	Folded Trajectories	Folding Time (ns)
SARS-CoV-1 RBD	0.9306	56	369.0	58	304.95
SARS-CoV-2 RBD	0.8745	97	257.4	42	5646.18

## Data Availability

The original contributions presented in the study are included in the article and supplementary material, further inquiries can be directed to the corresponding author.

## Supplementary Materials

The following supporting information can be downloaded at: https://www.mdpi.com/article/10.3390/biom14101327/s1, Figure S1: Metastable states in the folding process of SARS-CoV-1 RBD; Figure S2: Metastable states in the folding process of SARS-CoV-2 RBD.
