# Supplementary material for: Pulling Forces Differentially Affect Refolding Pathways Due to Entangled Misfolded States in SARS-CoV-1 and SARS-CoV-2 Receptor Binding Domain
Source: Biomolecules. 2024 Oct 18;14(10):1327. doi: 10.3390/biom14101327 (PMC11505858; doi:10.3390/biom14101327)
Supplement: Supplementary file 1 [file biomolecules-14-01327-s001.zip › biomolecules-3254314-supplementary.pdf]

Supplementary Information

Pulling Forces Differentially Affect Refolding Pathways Due to Entangled Misfolded States in SARS-CoV-1 and SARS-CoV-2 Receptor Binding Domain

Pham Dang Lan 1,2, Edward P. O'Brien 3,4,5 and Mai Suan Li 6,*

1 Institute for Computational Sciences and Technology,
Ho Chi Minh City 71506, Vietnam; l72mss@gmail.com
2 Faculty of Physics and Engineering Physics, VNUHCM-University of Science, 227, Nguyen Van Cu Street, District 5, Ho Chi Minh City 72700, Vietnam
3 Department of Chemistry, Pennsylvania State University,
University Park, PA 16802, USA; epo2@psu.edu
4 Bioinformatics and Genomics Graduate Program, The Huck Institutes of the Life Sciences, Pennsylvania State University, University Park, PA 16802, USA
5 Institute for Computational and Data Sciences, Pennsylvania State University, University Park, PA 16802, USA
6 Institute of Physics, Polish Academy of Sciences, 02-668 Warsaw, Poland
* Correspondence: masli@ifpan.edu.pl

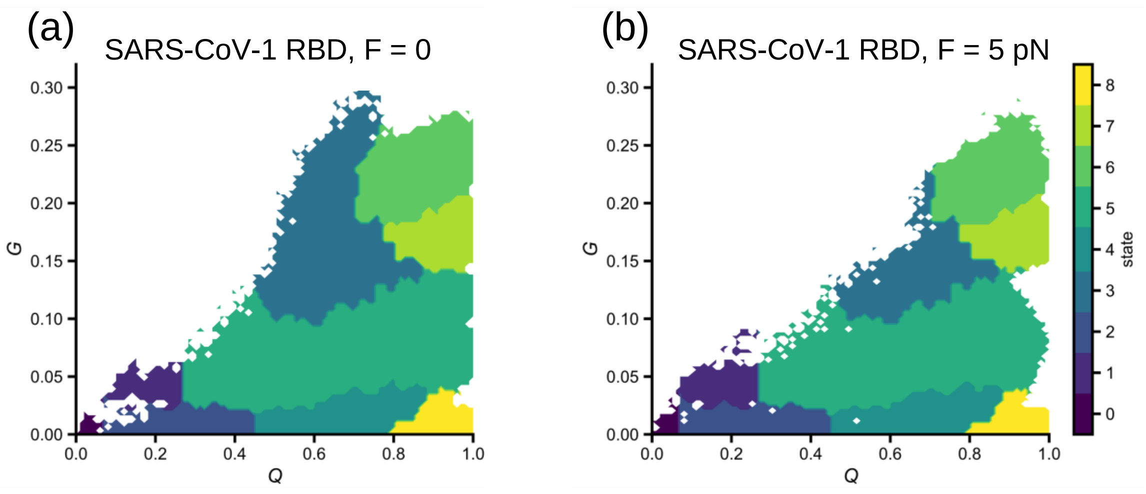

Figure S1. Metastable states in the folding process of SARS-CoV-1 RBD. Colored regions corresponding to different metastable states used to define folding pathways of SARS-CoV-1 RBD for (a) simulations with $F = 0$, and (b) simulations with $F = 5$ pN.

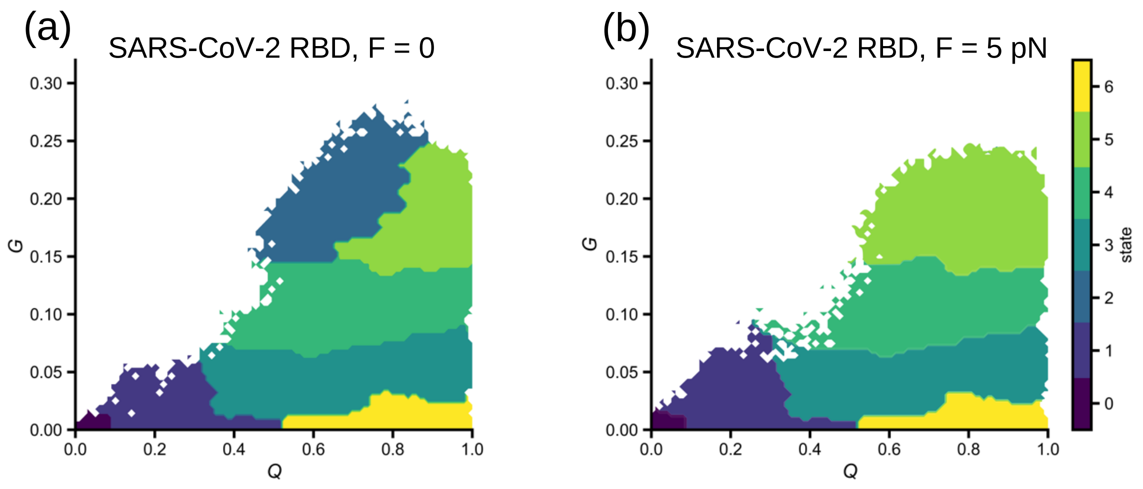

Figure S2. Metastable states in folding process of SARS-CoV-2 RBD. Colored regions corresponding to different metastable states used to define folding pathways of SARS-CoV-2 RBD for (a) simulations with $F = 0$, and (b) simulations with $F = 5$ pN.
